# Supplementary material for: The Kappa Opioid Receptor Agonist 16-Bromo Salvinorin A Has Anti-Cocaine Effects without Significant Effects on Locomotion, Food Reward, Learning and Memory, or Anxiety and Depressive-like Behaviors
Source: Molecules. 2023 Jun 19;28(12):4848. doi: 10.3390/molecules28124848 (PMC10304272; doi:10.3390/molecules28124848)
Supplement: Supplementary file 1 [file molecules-28-04848-s001.zip › molecules-2408394-supplementary.pdf]

Supplementary material for

The Kappa Opioid Receptor Agonist 16-Bromo Salvinorin A Has Anti-Cocaine Effects Without Significant Effects on Locomotion, Food Reward, Learning and Memory, or Anxiety and Depressive-like Behaviors.

Ross van de Wetering, Amy Ewald, Susan Welsh, Lindsay Kornberger, Samuel E. Williamson, Bryan D. McElroy, Eduardo R. Butelman, Thomas E. Prisinzano, and Bronwyn M. Kivell

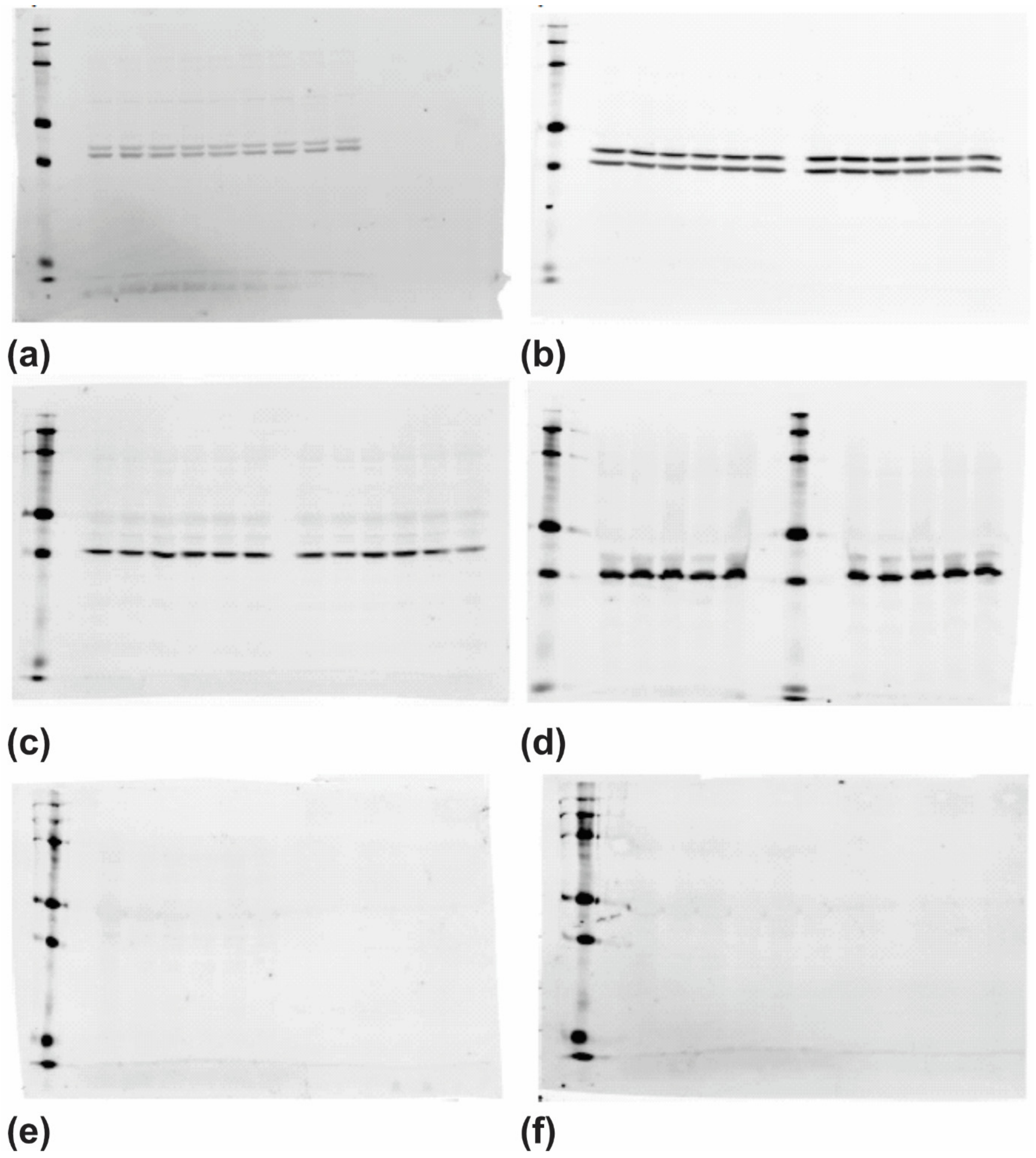

Figure S1. Representative western blot membranes for p-ERK1/2 (**a**), ERK1/2 (**b**), p-p38 (**c**), and p38 (**d**). Control blots showing non-specific binding of anti-mouse Cy5 (**e**) and anti-rabbit Cy5 (**f**).
